# Prophylactic and post-exposure efficacy of a Pichinde virus vector-based tuberculosis vaccine

**DOI:** 10.3389/fimmu.2026.1861052

**Published:** 2026-06-30

**Authors:** Michaela Cain, Alisha M. Block, Qinfeng Huang, Natalie M. Kirk, Hannah Murphy, Macallister C. Harris, Hinh Ly, Anna D. Tischler, Yuying Liang

**Affiliations:** 1 Department of Veterinary and Biomedical Sciences, College of Veterinary Medicine, University of Minnesota, St. Paul, MN, United States; 2 Department of Microbiology and Immunology, School of Medicine, University of Minnesota, Minneapolis, MN, United States; 3 Department of Microbiology, Immunology, and Pathology, College of Veterinary Medicine and Biomedical Sciences, Colorado State University, Fort Collins, CO, United States

**Keywords:** Mycobacterium tuberculosis, Pichinde virus, TB, vaccine, vaccine immunity, viral vector

## Abstract

*Mycobacterium tuberculosis* (*Mtb)* remains the leading cause of death from a single infectious agent, despite the availability of the Bacillus Calmette-Guérin (BCG) vaccine and antibiotic therapies. New tuberculosis (TB) vaccines are urgently needed to effectively protect adults against pulmonary *Mtb* infections, whether used alone, as a BCG booster, or as a therapeutic strategy. We developed TBpV1, a multivalent TB vaccine, using recombinant Pichinde virus (rPICV) vector rP18tri. TBpV1 comprises three rP18tri-vectored components that collectively deliver 13 known and novel antigens targeting both the latent and active stages of the *Mtb* infection cycle. In mice, the pooled TBpV1 vaccine elicited robust antigen-specific CD4^+^ and CD8^+^ T cell responses, including polyfunctional Th1 and IL-17-producing cells, at levels equivalent to individual rP18tri-vectored vaccines. As a standalone vaccine, TBpV1 significantly reduced bacterial burden following an *Mtb* Erdman aerosol challenge, achieving protection comparable to BCG. TBpV1 also enhanced protection when administered as a BCG booster, reducing lung inflammation and bacterial loads. Lastly, TBpV1 reduced splenic bacterial burden in a post-exposure setting. These results demonstrate that pooling multi-stage antigens via the rP18tri vector maintains immunogenicity while improving protective efficacy. TBpV1 is a promising candidate for both TB prevention and therapeutic intervention, supporting further evaluation in preclinical models.

## 1 Introduction

*Mycobacterium tuberculosis* (*Mtb)*, the causative agent of tuberculosis (TB), remains the world’s leading cause of death due to a single infectious agent despite the availability of antibiotic treatments and widespread use of the Bacillus Calmette-Guérin (BCG) vaccine in TB endemic areas (1). *Mtb* causes an estimated 10.7 million new cases of active disease and 1.23 million deaths annually and is the leading cause of death for individuals with HIV infection (1, 2). Following infection with *Mtb*, individuals have a 5-10% lifetime risk of developing active TB disease, and those with compromised immune systems have a higher risk of developing disease (3). Current antibiotic regimens require prolonged treatment courses of 6–9 months, and approximately 2-10% fail initial therapy or relapse (4). The emergence of multidrug-resistant TB (MDR-TB) and extensively drug-resistant TB (XDR-TB) further complicates treatment and represents a growing public health threat (2, 5). While the BCG vaccine provides protection against childhood forms of TB and reduces infant mortality, its variable efficacy against adult pulmonary TB highlights the need for improved vaccine strategies (6–8). The World Health Organization (WHO) TB vaccine advisory committee has emphasized the urgent need to develop improved TB vaccines that are effective across all age groups, in high-risk populations, and both pre- and post-exposure (9).

*Mtb* poses a unique challenge to vaccine development, due to its complex lifecycle and intricate interaction between the host immune system and bacterial virulence mechanisms (10, 11). The exact correlates of protection are unknown, although control of *Mtb* infection is thought to be heavily influenced by strong T-cell responses. IFN-γ and TNF-α produced by CD4^+^ Th1 cells act on infected macrophages and aid in the killing of *Mtb*, while CD8^+^ T cells mediate killing through the secretion of cytokines, perforin, and granzyme-granulysin pathways (12, 13). Recent studies show that protection against *Mtb* requires a complex balance between innate and adaptive immunity, but achieving such a goal through vaccination remains elusive (11, 14). Moreover, antigen availability during different stages of *Mtb* infection heavily impacts the efficacy of vaccine-generated immune responses (15). Most TB vaccine candidates use immunodominant antigens such as ESAT-6 and Ag85B, which are suboptimal at inducing protective immunity. Ag85B is only expressed during acute infection, and Ag85B-specific T cells do not effectively recognize *Mtb*-infected cells later in infection (15). ESAT-6 is produced throughout infection, but chronic exposure often drives these T cells toward a terminally differentiated, functionally exhausted state (15). As TB presents as both latent infection and active disease, protection against these distinct forms of disease may require different immune responses targeting diverse antigens, further complicating vaccine design and evaluation.

Our lab previously developed a recombinant Pichinde virus (rPICV) vaccine vector rP18tri (16, 17). PICV is a non-pathogenic arenavirus with no seroprevalence in the general human population (18). The rP18tri vector exhibits limited replication in the hosts, with no vector shedding reported for all tested animal species (16, 19, 20), and has been evaluated in preclinical models and Phase I clinical trials (NCT04180215) (21). Thus, the rP18tri vector demonstrates a favorable safety profile, which is important for use in immunocompromised populations (21). The rP18tri vector targets antigen-presenting cells (APCs), leading to strong activation of both antibody and T-cell responses (22, 23). A distinctive advantage of this rP18tri vector is the absence of detectable anti-vector neutralizing antibodies, even after repeated doses (24). In a previous study, we have shown that the rP18tri vector expressing model *Mtb* antigens induced robust systemic and tissue-resident antigen-specific CD4^+^ and CD8^+^ T cell responses following either intraperitoneal (IP) or intranasal (IN) administration (25), and significantly reduced bacterial burden in an *Mtb* aerosol challenge mouse model (25).

In the current study, we generated an rP18tri-based multivalent TB vaccine (TBpV1) delivering a panel of known and novel *Mtb* antigens. TBpV1 elicited broad and polyfunctional T-cell responses and provided significant protection against *Mtb* challenge in mice as a stand-alone vaccine, as a BCG booster, and as a post-exposure treatment.

## 2 Materials and methods

### 2.1 Ethics statements

Research conducted for this manuscript was approved by the Institutional Biosafety Committee at the University of Minnesota, Twin Cities, under the protocol IDs 2308-41352H and 2309-41371H. All animal procedures were approved by the Institutional Animal Care and Use Committee (IACUC) of the University of Minnesota, Twin Cities, under the protocol IDs 2304-40977A and 2302-40834A. Mouse anesthesia was induced by 4-5% isoflurane and maintained at 1-2% via anesthesia machine. Mice were euthanized with carbon dioxide (CO_2_) at a flow rate of 30-70% of the chamber volume per minute.

### 2.2 Mammalian cells, plasmids, viruses, and bacteria

BHK-21 Baby hamster kidney cells and Vero African green monkey kidney cells were grown in Dulbecco’s Modified Eagle’s Medium (DMEM) (Fisher Scientific) with 10% fetal bovine serum (FBS) (Sigma) and 50 μg/ml penicillin and streptomycin (Invitrogen-LifeTechnologies). BSRT7–5 cells, obtained from K.K. Conzelmann (Ludwig-Maximilians-Universität, Germany), are BHK-21 cells stably expressing T7 RNA polymerase. BSRT7–5 cells were grown in minimal essential medium (MEM) (Invitrogen-LifeTechnologies) with 10% FBS, 1 μg/ml Geneticin (Invitrogen-LifeTechnologies), and 50 μg/ml penicillin-streptomycin. Recombinant rP18tri viruses were amplified in BHK-21 cells and quantified by viral plaque assay in Vero cells as described previously (25). *Mtb* Erdman is a fully virulent isolate and a Biosafety Level 3 (BSL-3) agent. *Mtb* was routinely cultured at 37˚C with aeration in Middlebrook 7H9 liquid medium (Difco), which was supplemented with 10% albumin-dextrose-saline (ADS), 0.5% glycerol, and 0.1% Tween-80. *Mycobacterium bovis* Bacillus Calmette-Guérin (BCG) vaccine was cultured under the same conditions as *Mtb* Erdman but handled in a BSL-2 facility.

### 2.3 Proteins, peptides, tetramers, and antibodies

Peptide arrays of *Mtb* Ag85B (NR-34828) were obtained from BEI Resources. Pan-Tuberculosis BCG Select (PM-Pan-TBBCGselect-1) peptide arrays were purchased from JPT Peptide Technologies GmbH (Berlin, Germany). Anti-Ag85 Complex (NR-13800) antisera and polyclonal anti-*Mtb* whole cell lysate antisera (NR-1381) were obtained from BEI Resources.

Phycoerythrin (PE)-conjugated EsxH MHC-I tetramer (H-2K(b) IMYNYPAM), PE-labeled MHC-II Ag85B tetramer I-A(b)/FQDAYNAAGGHNAVF, and control MHC-II tetramer I-A(b)/PVSKMRMATPLLMQA were obtained from the NIH Tetramer Core Facility at Emory University. Antibodies used for T cell analysis of immunized mice were purchased from commercial sources, including allophycocyanin (APC)-labeled anti-CD3 (17A2) (Biolegend), fluorescein isothiocyanate (FITC)-labeled anti-CD4 (RM4-5) (Biolegend), peridinin chlorophyll protein (PerCP)-Cy5.5-labeled anti-CD8 (53-6.7) (eBioscience), brilliant violent (BV) 510-labelled anti-CD44 (IM7) (Biolegend), BV605-labeled CD62L (MEL-14) (BD Biosciences), BV421-labeled anti-TNFα (MP6-XT22) (Biolegend), phycoerythrin (PE)-labeled anti-IL-2 (JES6-5H4) (BD Biosciences), APC-labeled anti-IFNγ (XMG1.2) (BD Biosciences), and PE-labeled IL-17 (TC11-18H10.1) (Biolegend). Fixable viability Ghost Dye Red 710 (Cytek) was used to differentiate between live and dead cells. The secondary antibody used for confirmation of antigen expression was purchased from ThermoFisher, Goat anti-Rabbit IgG (H+L) Cross-Adsorbed Secondary Antibody, Alexa Fluor™ 488.

### 2.4 Generation of recombinant rP18tri-vectored vaccines encoding *Mtb* antigens

The protein sequences of *Mtb* antigens were obtained from GenBank. Gene fragments encoding single antigen or multiple antigens linked by a P2A linker sequence (GSGATNFSLLKQAGDVEENPGP) (26) were codon optimized for human cell expression and chemically synthesized by Twist Bioscience (South San Francisco, CA). These gene fragments were cloned into the S1 or S2 vector of the rP18tri reverse genetics system (16, 27). The resulting plasmids were verified by sequencing. Recombinant rP18tri-vectored vaccines were rescued, titered, and sequence confirmed as described previously (16, 24).

### 2.5 Detection of antigen expression by immunofluorescence assay

Vero cells grown on coverslips were mock-infected or infected with the rP18tri vector alone or respective rP18tri-vectored vaccines at a multiplicity of infection (MOI) of 0.1. At 24 hours post-infection (hpi), cells were fixed with 4% paraformaldehyde for 15 min at room temperature (RT), and washed three times with phosphate buffered saline (PBS) (Invitrogen-LifeTechnologies). Cells were treated with 0.1% Triton X-100 for 12 min, followed by incubation with rabbit polyclonal antibodies against *Mtb* whole cell lysate (BEI, NR-13819) for 1 h. After washing, cells were incubated with goat anti-rabbit IgG Alexa Fluor-488 (Invitrogen) for 1 hr at RT. The cells were washed three times and mounted on a glass slide for examination by confocal microscopy.

### 2.6 Mouse immunization and immune cell collection

C57BL/6 mice (*n* = 5) were immunized intraperitoneally (IP) or intranasally (IN) with a single dose of either PBS or 1x10^5^ plaque forming units (PFU) of rP18tri-vectored vaccines, based on our previously established immunization conditions (16). Peripheral blood mononuclear cells (PBMCs) or tissue lymphocytes were collected at 7 or 14 days after vaccination. Blood was collected via cardiac puncture into lithium heparin tubes (Greiner Bio-One). PBMCs were isolated by lysing red blood cells (RBCs) with 1x RBC lysis buffer (eBiosciences), followed by a wash step with RPMI-1640 supplemented with 10% FBS. Splenocytes were isolated by gently dissociating spleens through a 40-µm cell strainer and washed with RPMI-1640 medium containing L-glutamine (Cytiva HyClone). After lysing RBCs with 1x RBC lysis buffer, splenocytes were resuspended in RPMI-1640 with 10% FBS for ICS or PBS with 2% FBS for tetramer staining.

### 2.7 Evaluation of antigen-specific CD8^+^ T cells by MHC-I tetramer analysis

Isolated PBMCs were incubated with PE-labeled EsxH tetramer, fixable viability stain Ghost Dye Red 710, APC-labeled anti-CD3, PerCP-Cy5.5-labeled anti-CD8, FITC-labeled anti-CD4, BV510-labeled anti-CD44, BV605-labeled CD62L, and Purified Rat anti-Mouse CD16/CD32 Fc Block for 45 min on ice. Cells were then washed three times with PBS with 2% FBS. Sample acquisition was performed on a BD FACSCelesta Flow Cytometer and data analysis was performed using FlowJo (10.9).

### 2.8 Evaluation of antigen-specific CD4^+^ T cells by MHC-II tetramer analysis

Isolated PBMCs were incubated with PE-labeled Ag85B or PE-labeled MHC II Control tetramers, fixable viability stain Ghost Dye Red 710, APC-labeled anti-CD3, PerCP-Cy5.5-labeled anti-CD8, FITC-labeled anti-CD4, BV510-labeled anti-CD44, BV605-labeled CD62L, and Purified Rat anti-Mouse CD16/CD32 Fc Block for 45 min on ice. Cells were then washed three times with PBS with 2% FBS. Sample acquisition was performed on a BD FACSCelesta Flow Cytometer and data analysis was performed using FlowJo (10.9).

### 2.9 Intracellular cytokine staining

Isolated cells were seeded in complete RPMI-1640 with 10% FBS at 1x10^6^ cells per well and incubated at 37 °C with medium alone or Ag85B Peptide Array (NR-34828 BEI) in the presence of GolgiPlug (BD Biosciences) for 6 h. Cells were collected, washed once, resuspended in 400 µl of RPMI-1640 with 10% FBS, and incubated at 4 °C overnight. Cells were stained with surface markers FITC-CD4, PerCP-Cy5.5-CD8, BV510-CD44, and Fixable viability Ghost Dye Red 710 (Cytek). After being fixed and permeabilized using the Cytofix/Cytoperm kit (BD Biosciences), cells were incubated with cytokine antibodies for 40 min on ice: BV421-labeled anti-TNFα, PE-labeled anti-IL-2, and APC-labeled anti-IFNγ for Th1 T cells or PE-labeled anti-IL-17 for Th17 T cells. Sample acquisition was performed on FACSCelesta and data were analyzed using FlowJo.

### 2.10 IFNγ-based enzyme-linked immunosorbent spot assay

ELISpot was performed using ELISpot Flex: Mouse IFNγ (HRP) kit (3321-4HST-2, Mabtech) following the manufacturer’s instructions. Briefly, 96-well filtration plates (Millipore, S5EM077I10) were activated with 70% ethanol, washed with PBS, and coated with anti-mouse IFNγ capture antibody overnight at 4 °C. The following day, plates were washed and blocked with RPMI-1640 containing 10% FBS for 30 min at RT. 1x10^5^ splenocytes were added per well and stimulated with either medium only, Ag85B peptide pools (NR-34827, BEI), or pan-BCG peptide pools (PM-Pan-TBBCGselect-1, JPT) at 5 µg/ml for 18 h at 37 °C and 5% CO_2_. After incubation, a biotinylated anti-mouse IFNγ detection antibody was added and incubated overnight at 4 °C. Finally, streptavidin-Horseradish Peroxidase (HRP) and 3,3’,5,5’-Tetramethylbenzidine (TMB) were added for color development. Spots were recorded using Mabtech Astor2 ELISpot Reader and quantified using the Mabtech APEX software.

### 2.11 Evaluate vaccine-induced protection against *Mtb* aerosol challenge in mice

To evaluate stand-alone prophylactic protection by rP18tri-vectored vaccines, groups of six (n=3 females, n=3 males) BALB/c mice were immunized with 1x10^5^ PFU IP with TB10, TB23, TB26, or TBpV1, in a prime-boost regimen with a 21-d interval. As controls, one group of mice received PBS, and another received 1x10^6^ CFU of BCG (SC). The empty vector group, which did not show protection in our previous study (25), was omitted in this study to prioritize experimental groups. Two weeks post-boost, mice were challenged with ~100 CFU of virulent *Mtb* Erdman, using a Glas-Col Inhalation Exposure System, in a BSL-3 laboratory, as previously described (25). A group of unvaccinated mice (n=4) was euthanized at 24 h post-infection to confirm the *Mtb* infection. At 4 weeks post-infection, mice were sacrificed. The right lung lobes and spleen were aseptically collected for bacterial quantification, and the left lung lobe was collected for histopathological analysis.

To evaluate the protective efficacy of TBpV1 as a boost vaccine for BCG immunization, BALB/c mice (n=5) were primed with PBS (IP) or BCG (SC), and 21 d later, boosted (IP) with PBS or 1x10^5^ PFU of TBpV1 (IP or IN). Three weeks post-boost, mice were challenged with ~100 CFU of virulent *Mtb* Erdman as described above.

To evaluate post-exposure protection of TBpV1, BALB/c mice (n=6) were challenged with ~100 CFU of virulent *Mtb* Erdman as described above, and immunized (IP) with PBS or 1x10^5^ PFU of TBpV1 at 1- and 21-days post challenge. Mice were euthanized 5 weeks post challenge. Lungs and spleens were collected and analyzed as described above.

### 2.12 Histopathologic analysis

Lungs were removed aseptically at necropsy. The left lung of each mouse was immersed in 10% neutral buffered formalin for 24 hours and then transferred to 70% ethanol. Lungs were then embedded in paraffin, cut, and stained with hematoxylin and eosin (H&E). Slides were scanned at 40X using a Vectra Polaris scanning microscope. Images were imported into VisioPharm version 2025.08.1.18881 x64 and processed through an artificial intelligence (AI) workflow for quantitative evaluation of total lung inflammation, peribronchial and perivascular inflammation, and macrophage and lymphocyte populations. Quantification was performed using the whole lung tissue with output measurements reported as percentage of total lung area (total inflammation) or percentage of total inflammation (peribronchial inflammation, perivascular inflammation, macrophages, and lymphocytes). The workflow and applications were designed, annotated, trained, and verified by two board-certified veterinary anatomic pathologists. Briefly, the workflow consisted of stepwise applications with increasing specificity from identification of lung tissue, identification of airways and vessels, identification of inflammation, and, finally, classification of macrophages and lymphocytes in areas of inflammation. The results were confirmed manually at each step to ensure accurate cellular identification. Lung sections from intranasally vaccinated, noninfected mice did not show any residual inflammation from vaccination.

### 2.13 Statistical analysis

All statistical analyses were performed using GraphPad Prism 10.0.2. For comparison between groups, unpaired two-tailed t tests and a 95% confidence interval were performed. P-values of less than 0.05 were considered significant and P-values of less than 0.01 were considered highly significant.

## 3 Results

### 3.1 Generation of an rP18tri-vectored multivalent TB vaccine TBpV1

**Figure 1:**
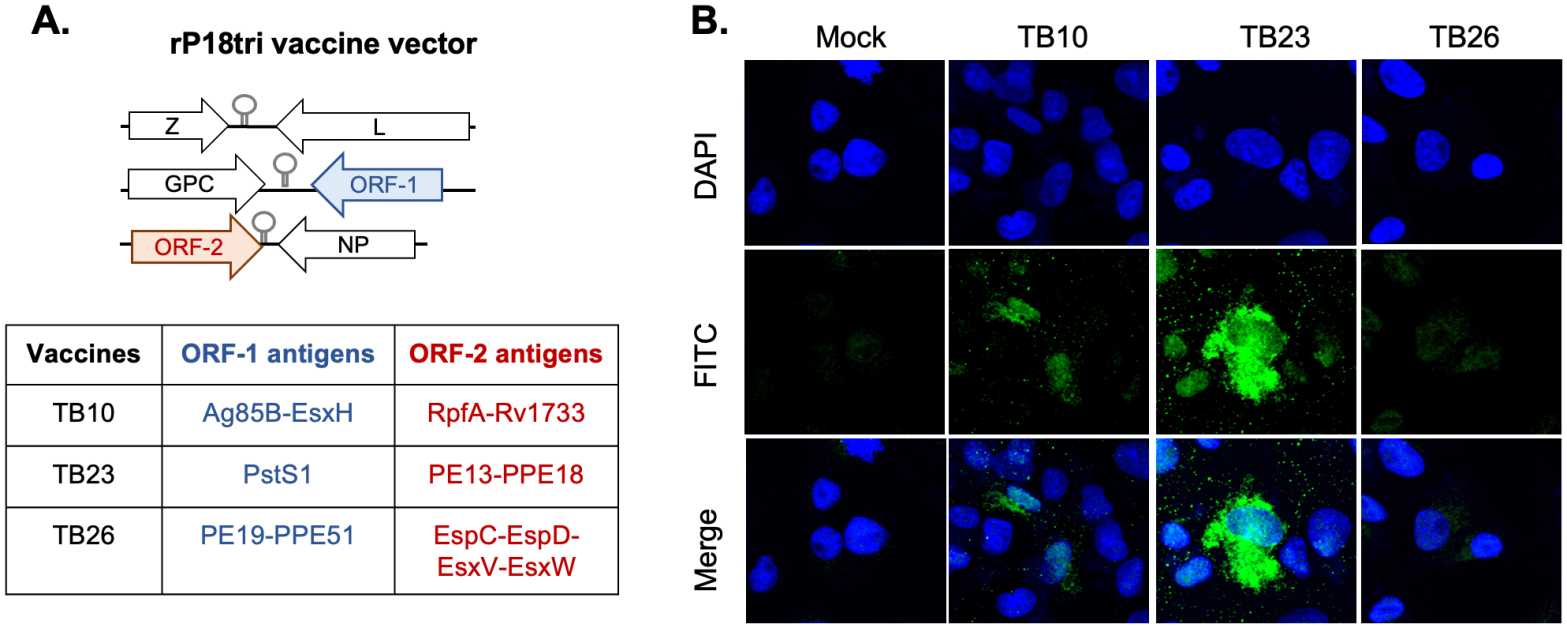
The multivalent TB vaccine, TBpV1, comprises three rP18tri-vectored vaccines, TB10, TB23, and TB26. **(A)** Illustration of the genomic organization of the rP18tri vaccine vector (top), with the encoded *Mtb* antigens shown for individual vaccines (bottom). **(B)** Detection of *Mtb* antigen expression in Vero cells infected with individual vector vaccines by IFA with *Mtb*-specific rabbit antisera and FITC-conjugated anti-rabbit IgG antibodies.

We previously generated rP18tri vector vaccines expressing model *Mtb* antigens, Ag85B, ESAT-6, and EsxH, which elicited strong systemic and lung T cell immunity with protective efficacy in mice (25). These well-defined *Mtb* antigens likely represent a small subset of T-cell targets. To improve the overall protective efficacy, we generated a multi-valent *Mtb* vaccine TBpV1, by using a combination of three rP18tri vector vaccines, TB10, TB23, and TB26, each encoding known and new *Mtb* antigens. The novel antigens include the lipoprotein PstS1 as well as proteins exported by the ESX-1 or ESX-5 type VII secretion systems and exposed on the *Mtb* cell surface. TB10, expressing Ag85B, EsxH, RpfA, and Rv1733, was previously constructed and evaluated for immune responses (25). TB23 and TB26 were generated in this study by the established reverse genetics system (16, 17). TB23 expresses PstS1, PE13, and PPE18, while TB26 expresses EspC, EspD, EsxV, EsxW, PE19, and PPE51 (**Figure 1A**). PE and PPE proteins, which form heterodimers for stable production and secretion (28–30), were paired together (PE19-PPE51 and PE13-PPE18) with a P2A linker in the same ORF. A P2A linker was also used to express EspC, EspD, EsxV, and EsxW from the same transcript on the S2 segment of TB26. Each vector vaccine has been confirmed by full-genome sequencing. Antigen expression was also confirmed by IFA using *Mtb*-specific rabbit antisera (**Figure 1B**). Strong signals were detected in cells infected with TB10 and TB23 but not with TB26, likely due to the variable levels of antigen-specific antibodies in the polyclonal serum.

### 3.2 Evaluation of vaccine-induced T-cell responses by ELISpot assay

**Figure 2:**
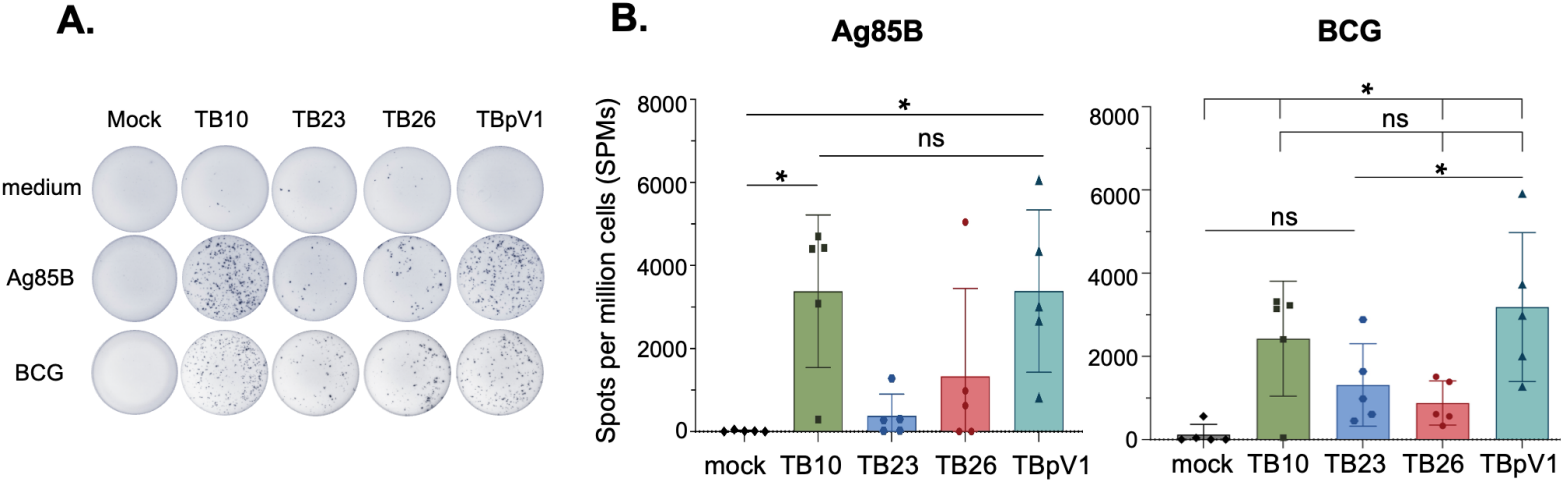
rP18tri-vectored TB vaccines induce antigen-specific T cells. C57BL/6 mice (*n=5*) were immunized (IP) once with PBS (mock) or the respective rP18tri-vectored vaccines. Splenocytes isolated 14 days post-vaccination (dpv) from mock or vaccinated mice were analyzed by IFN-γ ELISpot assay, following stimulation with medium only or peptide pools from Ag85B or BCG. **(A)** Representative image is shown for each group following stimulation with medium or peptide pools. **(B)** Normalized Spots Per Million (SPM) cells following stimulation with Ag85B (left) or BCG (right) peptide pools. ns, not statistically significant (*p* ≥ 0.05); *p < 0.05.

To evaluate antigen-specific T-cell responses, we conducted an ELISpot assay on splenocytes collected from C57BL/6 mice (*n* = 5) immunized with PBS (mock), single vector vaccines (TB10, TB23, and TB26), or the pooled vaccine TBpV1 at 14 days post vaccination (dpv). Splenocytes were stimulated with medium alone or with peptide panels for Ag85B or BCG (**Figure 2**). Compared to controls, the vaccine groups containing the Ag85B antigen (TB10 and TBpV1) showed a significantly increased number of IFNγ-secreting cells after stimulation with Ag85B peptides. Stimulation with pan-BCG peptides increased the number of IFNγ-secreting cells in all vaccine groups, with statistically significant differences observed for TB10, TB26, and TBpV1 (**Figure 2**). Pan-BCG peptides comprise 150 peptides from selected BCG proteins that are highly conserved with *Mtb* and include 7/13 encoded vaccine antigens (Ag85B, EsxH, PstS1, PE13, PPE18, PE19, PPE51). The robust pan-BCG responses induced by TBpV1 highlight its broad T-cell immune responses against *Mtb* antigens. Importantly, TBpV1 elicited responses to Ag85B- and BCG-specific peptide pools comparable to the TB10 vaccine, indicating no loss of T-cell immunogenicity in the pooled format.

### 3.3 TBpV1 elicits robust antigen-specific CD4^+^ and CD8^+^ T-cell responses

**Figure 3:**
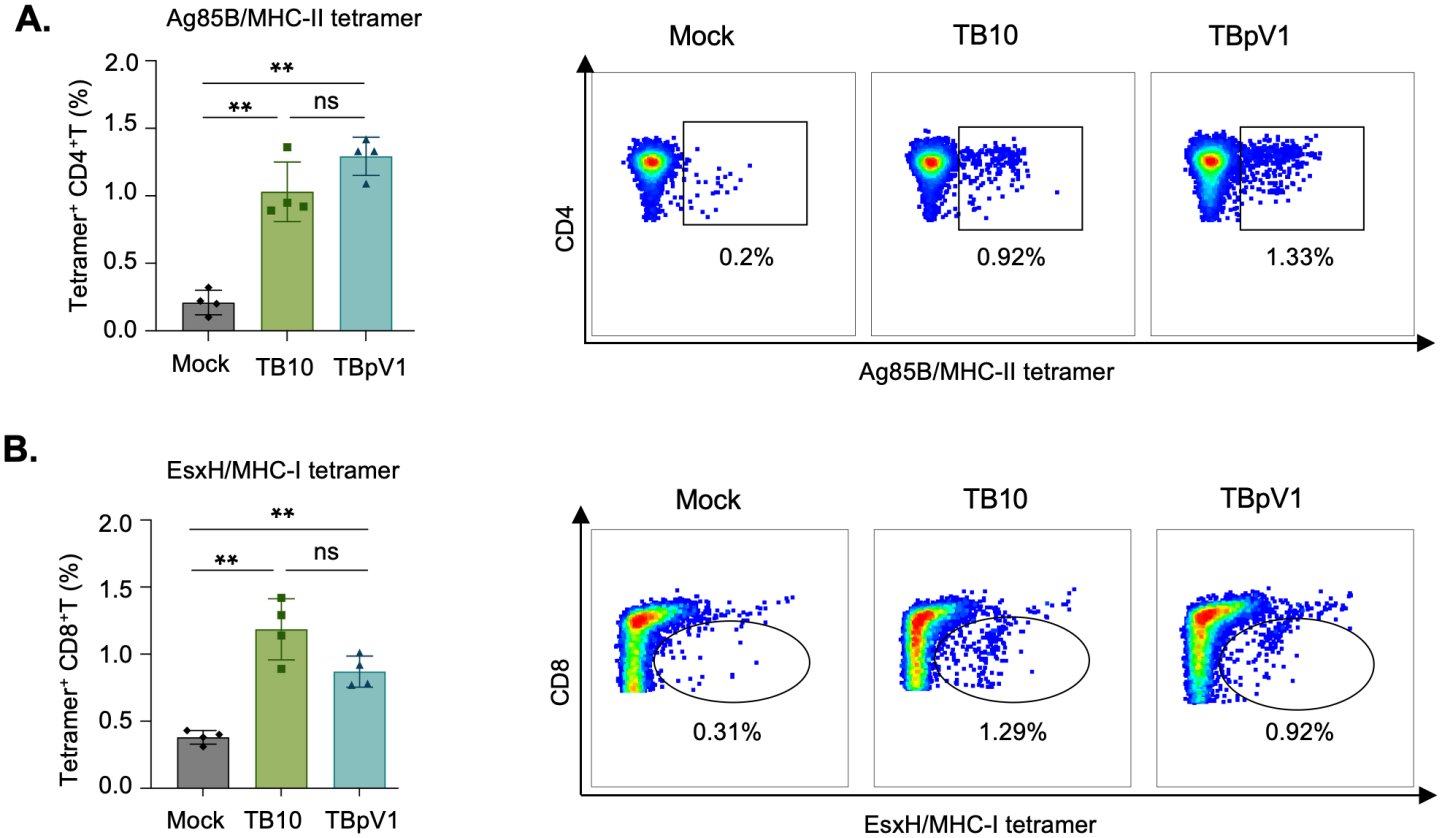
The pooled TBpV1 vaccine elicited comparable epitope-specific T cells as the individual TB10 vaccine. C57BL/6 mice (*n=4)* were immunized (IP) once with either PBS (mock), TB10, or TBpV1. **(A)** Splenocytes at 7 dpv were analyzed by Ag85B/MHC-II tetramer staining. Percentages of tetramer-positive CD4^+^ T cells are shown for each group (left) with representative flow cytometry plots (right). **(B)** PBMCs at 7 dpv were analyzed by EsxH/MHC-I tetramer staining. Percentages of tetramer-positive CD8^+^ T cells are shown for each group (left) with representative flow cytometry plots (right). ns, not statistically significant (*p* ≥ 0.05); **p < 0.01.

To assess CD4^+^ and CD8^+^ T-cell responses to the pooled vaccine TBpV1 compared to a single vaccine, C57BL/6 mice (*n* = 4) were immunized with PBS (mock), TB10, and TBpV1, respectively. Splenocytes collected at 7 d post-immunization were analyzed by the established Ag85B MHC-II tetramer assay to evaluate antigen-specific CD4^+^ T cells (**Figure 3A**) and the EsxH MHC-I tetramer assay to evaluate antigen-specific CD8^+^ T cells (**Figure 3B**). The gating strategy is shown in **Supplementary Figure 1**. Compared with mock immunization, both TB10 and TBpV1 induced significantly higher and comparable numbers of tetramer-positive CD4^+^ (**Figure 3A**) and CD8^+^ T cells (**Figure 3B**), suggesting that rP18tri-vectored vaccines elicit robust antigen-specific CD4^+^ and CD8^+^ responses without interference between individual vaccines when used in a pool.

### 3.4 TB10 and TBpV1 induce polyfunctional, antigen-specific CD4^+^ T cells in the spleen

**Figure 4:**
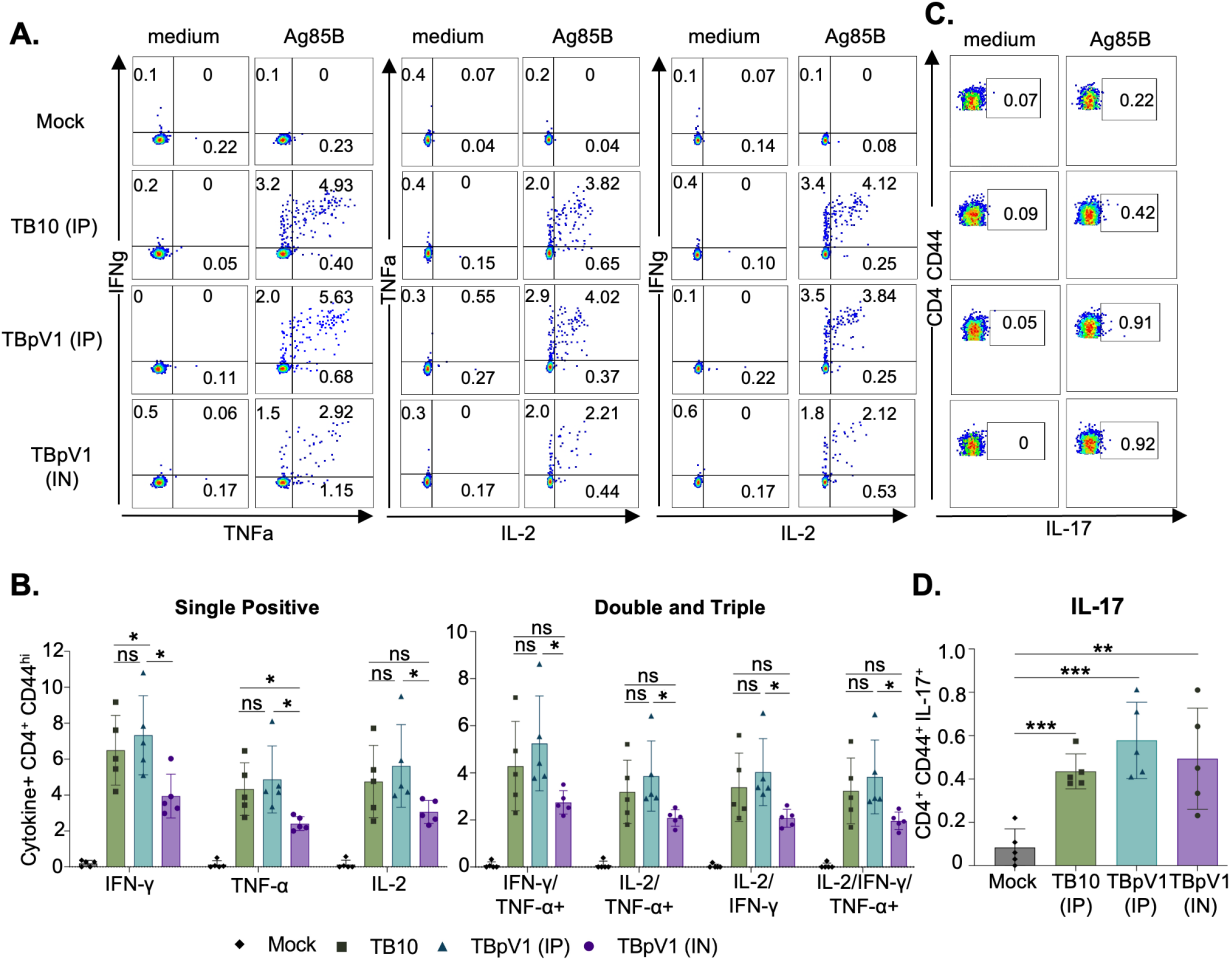
Polyfunctional antigen-specific CD4^+^ T cells and Th17 cells were elicited by TBpV1 and TB10 at comparable levels. Mice (*n=5)* were vaccinated once with PBS (mock), TB10 (IP), or TBpV1 (IN or IP). Splenocytes at 14 dpv were analyzed by ICS following stimulation with medium only or Ag85B peptide pools. **(A)** Representative flow cytometry plots of TNF-α/IFN-γ, IL-2/IFN-γ, and IL-2/TNF-α double-positive CD4^+^ T cells are shown for each group following stimulation with medium or Ag85B peptides. **(B)** Cytokine-producing single-, double-, and triple-positive CD4^+^CD44^hi^ T cells are shown for each group. **(C)** Representative flow cytometry plots of IL-17^+^ CD4^+^CD44^hi^ T cells are shown for each group following stimulation with medium or Ag85B peptides. **(D)** Percentages of IL-17-positive CD4^+^ CD44^hi^ cells were plotted for each group. All vaccine groups were significantly different from the mock group for single-, double-, and triple-positive cytokine secretion with *p* < 0.01. Significance values: ns, *p* ≥ 0.05; *p < 0.05, **p<0.01; ***p<0.001.

To evaluate the functionality of vaccine-induced T cells, C57BL/6 mice (n=5) were immunized with PBS (mock), TB10, or TBpV1 administered IP or IN. Splenocytes were collected 14 dpv, stimulated with Ag85B-specific peptides, which are 13 or 15 mers recognized by CD4^+^ T cells, for 6 h, and stained for cell surface markers and intracellular cytokines (IFNγ, IL-2, IL-17, and TNFα), and analyzed by flow cytometry to quantify CD4^+^ CD44^hi^ T cells producing one, two, and three cytokines (**Figure 4A**). The gating strategy is shown in **Supplementary Figure 2**. All vaccines induced significantly higher frequencies of cytokine-producing CD4^+^ T cells compared with the mock group (*p* < 0.002) (**Figure 4B**). TBpV1 IP vaccination induced the highest frequency of single-, double- and triple-positive CD4 CD44^hi^ T cells, followed by TB10 and TBpV1 administered IN (**Figure 4B**). Additionally, all vaccines induced significant CD4 CD44^hi^ IL-17-secreting T cells compared to mock vaccinated animals (**Figure 4C, D**). These data suggest that rP18tri-based TB vaccines stimulate antigen-specific Th17 CD4^+^ T cells and antigen-specific polyfunctional Th1 CD4^+^ T cells.

### 3.5 TBpV1 as a standalone prophylactic vaccine reduced bacterial load in mouse lungs and spleens in a virulent *Mtb* infection model

**Figure 5:**
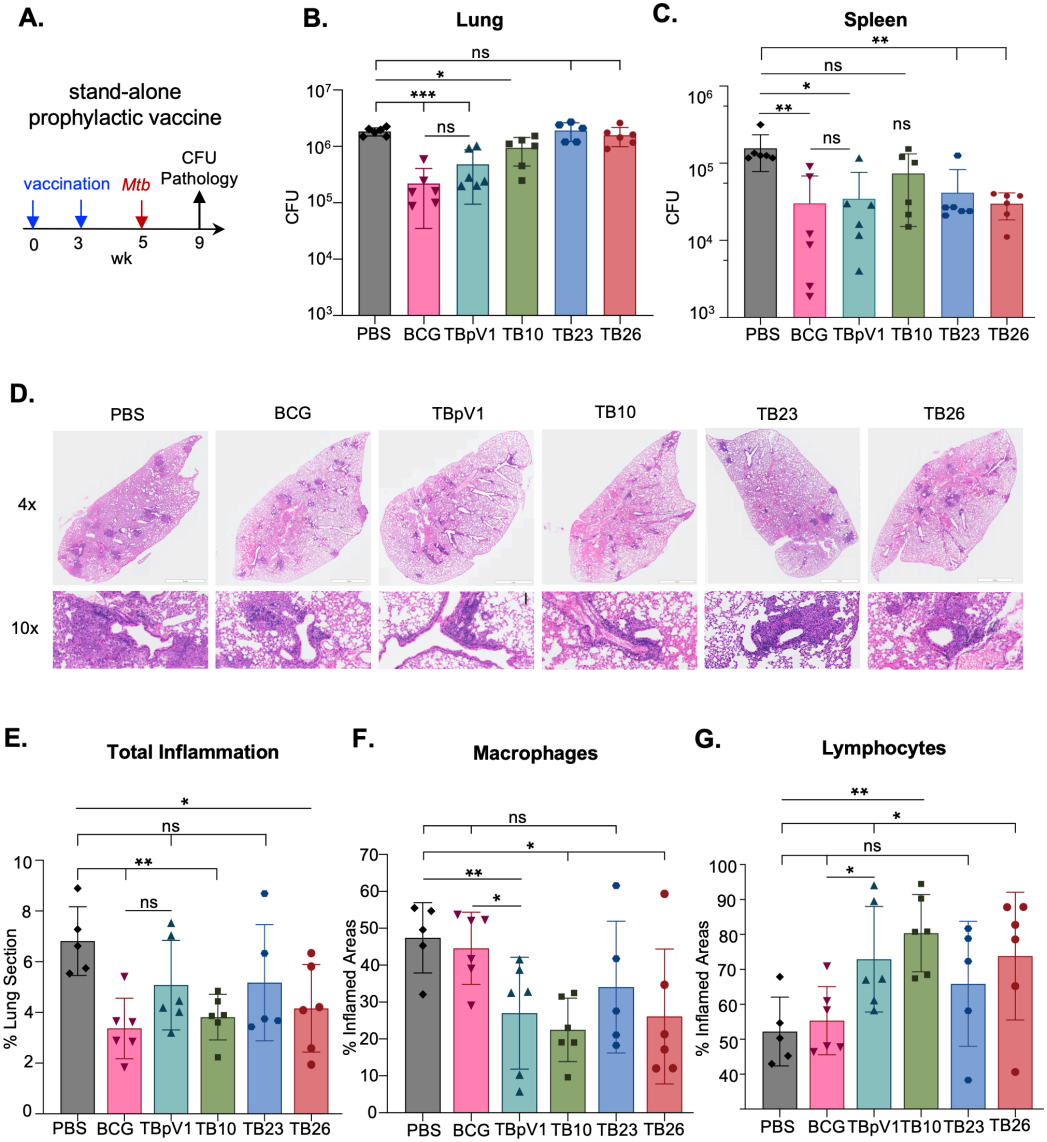
The protective efficacy of rP18tri-vectored TB vaccines as a stand-alone prophylactic vaccine in a virulent *Mtb* infection model. **(A)** Schematic illustration of experimental design. BALB/c mice *(n=*6*)* were vaccinated with either PBS (IP), a single dose of BCG (SC), or two doses of rP18tri-vectored vaccines (IP) before challenge with *Mtb* Erdman. Bacterial burdens (CFU) in lungs **(B)** and spleens **(C)** at 4 weeks post-challenge are shown for each group. Data represent mean ± SEM. **(D)** Representative H&E–stained sections of the left lung lobe imaged at 4× and 10× magnification. **(E)** Quantification of total inflammation, **(F)** macrophage infiltration, **(G)** and lymphocyte infiltration in inflamed lung areas. Statistical significance was determined using Welch’s t-test, ns, *p* ≥ 0.05; *p < 0.05; **p < 0.01; ***p < 0.001.

To evaluate the prophylactic protection as a stand-alone vaccine, mice (n = 6) were immunized (IP) with individual (TB10, TB23, and TB26) or pooled (TBpV1) vector vaccines, along with PBS and BCG controls, and 14 d after vaccination, challenged with a virulent *Mtb* strain through aerosol infection (**Figure 5A**). Bacterial load in mouse lungs and spleens at 4 weeks after challenge was measured by CFU (**Figure 5B, C**). Among the single vector vaccines, TB10 significantly reduced lung CFUs, while TB23 and TB26 reduced bacterial CFU in the spleens relative to the PBS control (**Figure 5C**). Both BCG and the pooled TBpV1 vaccine significantly reduced bacterial loads in both organs to comparable levels, demonstrating protective efficacy against *Mtb* replication in the lungs and subsequent dissemination to the spleen (**Figure 5B, C**).

Lung histopathology was analyzed by H&E staining (**Figure 5D**). Infected lungs had numerous infiltrates of epithelioid macrophages and lymphocytes that formed peribronchiolar and perivascular nodular aggregates. Bronchus-associated lymphoid tissue (BALT) was increased. This tissue inflammation was quantified using VisioPharm AI software for each animal as the percentage of total inflammation normalized to tissue area and shown as the average with standard deviation for each group (**Figure 5E**). Compared to the control group, vaccination with BCG, TB10, and TB26 significantly reduced lung inflammation. While TB23 and TBpV1 also showed a trend toward reduced inflammation, these differences did not reach statistical significance, likely due to the limited sample size and the high degree of variability (**Figure 5E**). These data suggest that rP18tri-vectored vaccines can confer protection against *Mtb*-induced lung pathology, even if lung bacterial loads are not significantly reduced, such as with the TB26 vaccine.

Infiltrated macrophages (**Figure 5F**) and lymphocytes (**Figure 5G**) within the inflamed regions were also quantified using VisioPharm AI software. Compared to mock and BCG, all rP18tri-vectored vaccines (TB10, TB23, TB26, and TBpV1) resulted in reduced macrophage and increased lymphocyte infiltration after *Mtb* challenge, although not all comparisons reached statistical significance. Whether these changes in the infiltrated cell composition correlate with lung bacterial load reduction requires further studies.

### 3.6 The TBpV1 vaccine further decreased lung bacterial load and reduced lung pathology when given as a BCG booster

**Figure 6:**
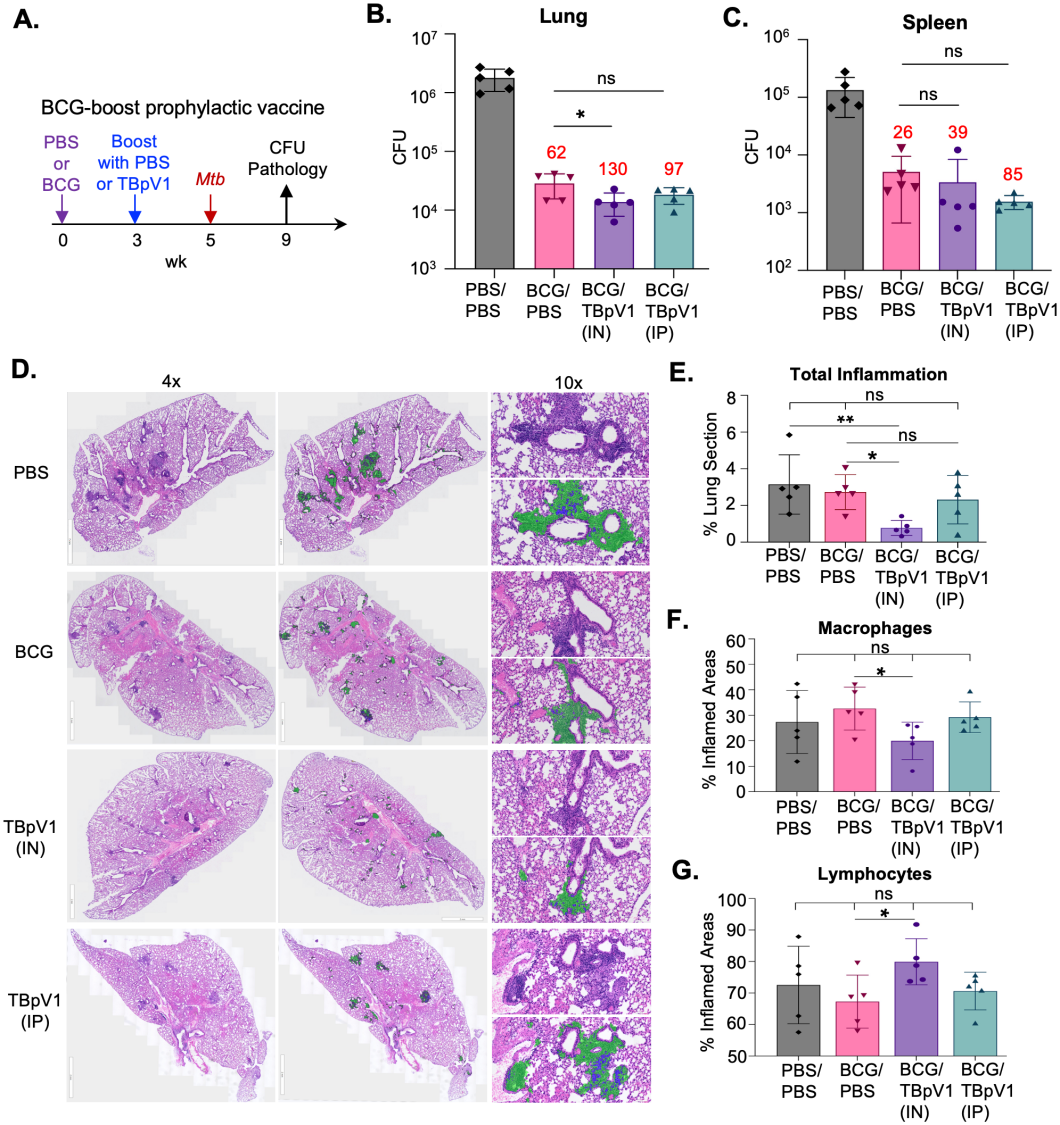
The protective efficacy of TBpV1 as a BCG booster vaccine in a virulent *Mtb* infection model. **(A)** Schematic illustration of experimental design. BALB/c mice (*n=5)* were primed with PBS or BCG (SC) and boosted with PBS or TBpV1 (IN or IP) before challenge with *Mtb* Erdman. Bacterial burdens (CFU) in lungs **(B)** and spleens **(C)** at 4 weeks post-challenge are shown for each group. Data represent mean ± SEM. Fold reduction of CFU compared to PBS is shown in red. **(D)** Representative H&E–stained sections of the left lung lobe imaged at 4× and 10× magnification. Macrophages in inflamed areas are highlighted in blue and lymphocytes in green. **(E)** Percentages of total inflammation in the lung sections are shown for each group. Macrophage **(F)** and lymphocyte **(G)** infiltration in the inflamed lung areas are shown for each group. Statistical significance was determined using Welch’s t-test, *p* ≥ 0.05; *p < 0.05; **p < 0.01.

BCG is generally given to all healthy neonates shortly after birth in TB-endemic areas and to high-risk individuals in areas with low TB incidence (7, 31). As such, newly developed TB vaccines are most likely to be used in BCG-immunized people to provide enhanced protection against adult pulmonary TB. We therefore evaluated the protective efficacy of TBpV1 as a BCG boost vaccine in the *Mtb* aerosol infection mouse model (**Figure 6A**). Mice (*n* = 5) were immunized with BCG (SC) and boosted with either PBS or TBpV1 via the IN or IP route. Bacterial CFUs were determined for the lung (**Figure 6B**) and spleen (**Figure 6C**) at 4 weeks post-challenge. Compared to mock, BCG alone significantly reduced the bacterial loads in lungs (62-fold reduction) and spleens (26-fold). TBpV1 boosting caused a greater reduction of bacterial burdens in both the lungs (130-fold via the IN route and 97-fold via the IP route) and spleens (39-fold via the IN route and 85-fold via the IP route), though only lung CFU after TBpV1 (IN) boosting reached statistical significance when compared to BCG alone.

Histopathological analysis of the lung sections was conducted by H&E staining, with macrophages in inflamed areas highlighted in blue, and lymphocytes highlighted in green (**Figure 6D**). The percentage of inflamed lung area was significantly reduced in mice boosted with TBpV1 (IN) compared to those receiving BCG alone (**Figure 6E**), consistent with the significantly decreased lung *Mtb* burden observed in this group (**Figure 6B**). Initial screening identified changes among the groups in the number of infiltrated macrophages and lymphocytes, but not neutrophils. We thus quantified infiltrated macrophages (**Figure 6F**) and lymphocytes (**Figure 6G**) within inflamed regions. Compared to BCG alone, IN boosting with TBpV1 demonstrated a statistically significant reduction in macrophage infiltration and an increase in lymphocyte infiltration. This shift in cellular composition, associated with reduced lung bacterial burden and decreased lung inflammation, suggests that TBpV1(IN)-induced T cells may promote the targeted clearance of *Mtb-*infected macrophages (32, 33). Taken together, our data demonstrate TBpV1 boosting, particularly via the IN route, augments the protective efficacy of BCG vaccination by reducing both bacterial burden and lung pathology.

### 3.7 The TBpV1 vaccine decreased bacterial load in the mouse spleen when given post-exposure

**Figure 7:**
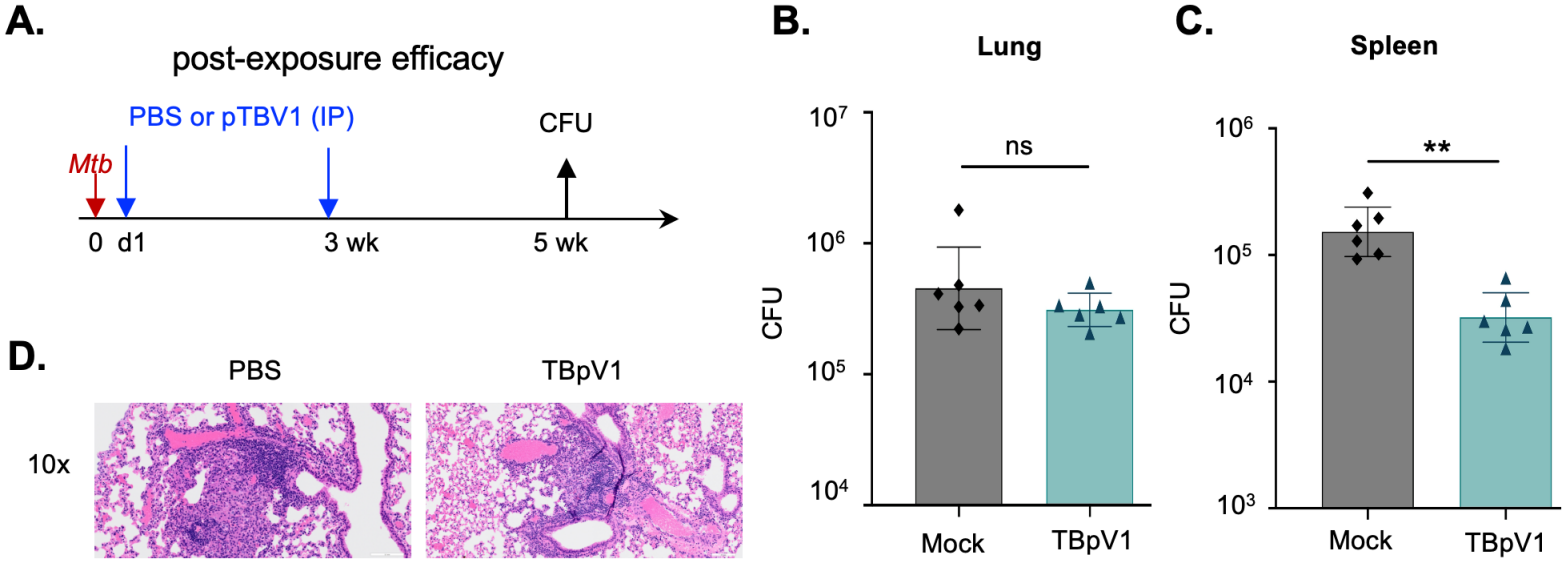
Efficacy of TBpV1 given post-exposure in a virulent *Mtb* infection model. **(A)** Schematic illustration of experimental design. BALB/c (*n=6)* were infected with *Mtb* Erdman and treated with either PBS or TBpV1 (IP) on day 1 and day 21 (3 wk) after challenge. Bacterial burdens (CFU) in lungs **(B)** and spleens **(C)** at 5 weeks after challenge are shown for PBS (mock) and TBpV1-treated groups. **(D)** Representative H&E–stained sections of the left lung lobe imaged at 10× magnification. Data represent mean ± SEM. ns, *p* ≥ 0.05; **p < 0.01.

TB vaccines can also be used post-exposure, to limit disease progression or prevent the development of active TB in individuals with known *Mtb* exposure (34, 35). To evaluate whether TBpV1 can protect against *Mtb* infection after exposure, BALB/c mice (*n* = 6) were aerosol-infected with *Mtb* Erdman and administered (IP) either PBS or pTBV1 on days 1 and 21. Bacterial CFUs in the lungs and spleens were quantified on day 35 (**Figure 7A**). Compared to PBS (mock), TBpV1 vaccination significantly reduced bacterial burden in the spleen, but not in the lung (**Figure 7B, C**). Inflammation in the lungs was comparable between PBS- and TBpV1-treated mice, consistent with the lack of reduction in CFU (**Figure 7D**). Together, these data suggest that systemic administration of TBpV1 can limit *Mtb* dissemination to peripheral organs but has limited efficacy in controlling *Mtb* replication in the lungs post-exposure.

## 4 Discussion

In this study, we evaluated the immunogenicity and efficacy of three rP18tri-vectored vaccines (TB10, TB23, and TB26), tested individually and as a pooled formulation in mice. The pooled vaccine TBpV1 collectively delivers 13 known and novel *Mtb* antigens and elicited antigen-specific CD4^+^ and CD8^+^ T cells at a comparable level to individual vaccines. All these rP18tri-vectored vaccines induce polyfunctional CD4^+^ T cells expressing Th1 cytokines (IFN-γ, IL-2, and TNF-α) and IL-17. TBpV1 provided greater protection than the individual vaccines and performed comparably to BCG against a virulent *Mtb* aerosol challenge in mice when tested as a stand-alone prophylactic vaccine. TBpV1 also enhanced protection when used as a BCG booster and reduced splenic bacterial burden when administered after infection. These results support the further evaluation and development of TBpV1 as a lead vaccine candidate for TB prevention and treatment.

Our data show that pooling rP18tri-vectored vaccines enables broader antigen delivery while maintaining immunogenicity of individual antigens, leading to improved protection. This is consistent with previous studies that expanding antigen selection and combining antigen families in a single dose enhances immune diversity and vaccine efficacy (36–39). Antigens in TBpV1 were selected based on immunogenicity in humans and mice, expression during acute infection and reactivation from latency, and predicted secretion or cell-surface localization. Ag85B and EsxH/TB10.4 are well-characterized immunodominant antigens primarily produced during the early exponential phase of bacterial growth and have demonstrated protection in various animal models as components of many vaccine candidates (40–42). The phosphate transport receptor PstS1 is a strong immunogen capable of inducing Th1 and Th17 immunity (43, 44), and human PstS1 antibodies can reduce bacterial burden in an *Mtb* aerosol challenge mouse model (45). TBpV1 includes two PE and PPE protein pairs, PE19/PPE51 and PE13/PPE18, which form heterodimers for stable production and secretion (30, 46, 47). These PE and PPE proteins are secreted by the ESX-5 type VII secretion system (48, 49), localize to the *Mtb* cell surface (46, 50), and are CD4^+^ T cell targets in humans (51, 52) and mice (53–55). PPE18 is included in a subunit vaccine M72/AS01E (56), which showed 50% efficacy at preventing active TB disease in *Mtb*-infected adults in a Phase IIb clinical trial (57). When tested as a single antigen in a DNA vaccine, PE19, PPE51, and PPE18 are among the top 20 antigens, conferring > 1.5-fold protection in mice (36). In addition, several Esp and Esx proteins, EspC, EspD, EsxV, and EsxW, were included in TBpV1 as potent human T-cell antigens (54, 58). EspC, EsxV, and EsxW have been used in fusion protein vaccines with demonstrated protection in mice (59–61). As the ESX-5 type VII secretion system is induced in acutely infected mice (62) and during resuscitation from hypoxia-induced dormancy (63), ESX-5 secreted antigens are likely to be available during both initiation of *Mtb* infection and reactivation from latency. In fact, we have shown that PE19 is required for chronic persistence of *Mtb* in C57BL/6 mice (64). EsxV and EsxW were included in the subunit vaccine ID93-GLA-SE that enhanced the efficacy of antibiotic treatment against tuberculosis in Cynomolgus Macaques (65). Lastly, TBpV1 includes two latency-associated proteins, RpfA and Rv1733c, which were components of a rhesus cytomegalovirus vector-based TB vaccine RhCMV/TB that has shown long-term efficacy for TB prevention in the rhesus macaque model (66). Given the presence of multiple protective antigens, it is not surprising that TBpV1 induces significant protection in mice. However, this study only evaluated the efficacy in an active tuberculosis mouse model. Additional testing in latent TB infection models is required to determine whether TBpV1 can also prevent *Mtb* reactivation (35, 67).

The strong polyfunctional, antigen-specific CD4^+^ and CD8^+^ T cell responses elicited by rP18tri-vectored TB vaccines highlight the platform’s ability to generate potent cellular immune responses, which are essential for effective TB vaccines (68, 69). Traditionally, robust CD8^+^ T cell responses require APCs to acquire exogenous viruses and cross-present antigens on MHC-I molecules following proteome-mediated degradation of viral proteins (70). In contrast, PICV preferentially infects dendritic cells (DCs) (22, 23), enabling direct presentation of intracellularly synthesized viral proteins on MHC-I (23). This results in high levels of antigen display and drives a strong cytotoxic lymphocyte (CTL) response (16, 24, 71). The sustained antigen presentation in cells infected with the replication-competent rP18tri vector also likely contributes to the magnitude of both CD4^+^ and CD8^+^ T cell responses.

Th17-polarized CD4^+^ T cells have emerged as a potential contributor of protective immunity against *Mtb*, although their exact role remains unclear (72–74). Notably, both TB10 and TBpV1 induced IL-17-producing CD44^hi^CD4^+^ T cells, which have been implicated in protective immunity through promotion of memory responses, granuloma formation, and acceleration of pathogen clearance in mice (75, 76). In humans, IL-17-secreting T cells correlate with enhanced control of *Mtb*, both during early clearance following exposure, and in preventing progression to active TB among individuals with latent TB (77). Subunit vaccines often require repeated dosing and Th17-promoting adjuvants, such as alum or CAF01, to elicit IL-17 responses (69, 78), while among viral vectors, MVA85A has been shown to induce IL-17 production in humans (79). Our results, alongside other vaccine studies, suggest that Th17 CD4^+^ T cells may be leveraged to enhance vaccine efficacy.

Our data demonstrate that TBpV1 provides prophylactic protection both as a stand-alone vaccine and as a BCG booster, as evidenced by both decreased bacterial burdens and reduced lung pathology. Compared to other advanced TB vaccines, TBpV1 demonstrates similar or improved efficacy in *Mtb* aerosol mouse challenge models. The subunit vaccine CAF^®^10b with adjuvant, currently in Phase 1 clinical trials, consists of a fusion protein of eight *Mtb* antigens with a novel adjuvant and conferred protection in CB6F1 mice challenged with aerosolized *Mtb* Erdman after three standalone or two BCG-boost doses (61). TBpV1 achieved comparable protection in BALB/c mouse model, as both a standalone vaccine and a BCG booster, with fewer vaccination doses, highlighting its potency and dosing efficacy. Among viral-vectored vaccines, both MVA85A (Modified Vaccine Ankara expressing Ag85A) and ChAdOx1.85A (chimpanzee adenovirus expressing Ag85A) have advanced to phase II clinical trials and have shown moderate protection in BALB/c mice challenged with aerosolized *Mtb* H37Rv or Erdman (80, 81). TBpV1 achieved comparable, if not greater, reductions in *Mtb* burden in mice relative to ChAdOx1.85A (administered alone or as a BCG boost), MVA85A, and the heterologous MVA85A-ChAdOx1.85A prime-boost regimen (80, 81). Thus, our multi-antigen rP18tri platform confers protective efficacy comparable to leading subunit-adjuvant and viral vectored TB vaccines, while requiring markedly lower doses and avoiding the induction of anti-vector neutralizing antibodies (24).

Importantly, our post-exposure study provides encouraging evidence for the efficacy of rP18tri-vectored TB vaccines as a post-exposure therapy, suggesting that this platform may partially overcome *Mtb*-induced suppression of the adaptive immune response (82). Protection with TBpV1 was modest, with a significant reduction in *Mtb* burden observed in the spleen, while lung CFU and associated pathology were unchanged. The observed protection could be due to prevention of bacterial dissemination from the lungs to the spleen, enhanced immune responses within the spleen, or a combination of both mechanisms. Further optimization of antigen selection, dosing and timing may enhance its therapeutic utility. Future studies should explore the use of rP18tri-vectored TB vaccines as a treatment for latent infection and in combination therapy to enhance bacterial clearance and shorten antibiotic treatment duration, an especially important consideration in the context of emerging antibiotic-resistant *Mtb* strains.

Our data suggest that TBpV1 is a promising candidate for both TB prevention and therapeutic intervention, though optimizing the vaccine regimen, such as vaccine titer, dosing, and routes is required to maximize protection. This study has several limitations that warrant further investigation. First, the ICS analysis only evaluated a small subset of antigen-specific CD4 T cells using peptides from a single antigen Ag85B. Future studies using Tuberculin Purified Protein Derivative (PPD) or a pool of *Mtb* proteins as *in vitro* stimulants will provide a more comprehensive assessment of TBpV1-induced cellular immunity. Second, lung tissue-resident antigen-specific CD4^+^ and CD8^+^ T cells were not analyzed. We previously demonstrated that IN inoculation with rP18tri-vectored TB vaccines elicits high levels of antigen-specific CD4^+^ and CD8^+^ T cells in the lungs (25). Comparing the lung CD4^+^ and CD8^+^ T cells induced by TBpV1 (IP and IN) or BCG, and correlating them with protection against pulmonary bacterial burden, will provide important insights to protective immunity. Third, this study focused on vaccine-induced effector T cells rather than memory T cells. While effector T cells may function in post-exposure protection, memory T cells are important for long-term prophylactic protection. Previously we showed that the rPICV-vectored vaccines elicit strong memory T cells that correlate with effector T cells (24). Thus, the high levels of effector T cells observed in this study likely suggest a robust memory T-cell response. Future studies will examine vaccine-induced, long-lived memory T cells, especially the phenotypic characterization of T-cell subsets using activation, effector, and exhaustion markers. This immunological profiling, combined with an evaluation of the long-term protection of TBpV1 will provide mechanistic insights into TBpV1-mediated protection and support its potential to extend BCG’s long-term efficacy. Fourth, vaccine-induced antibody responses were not evaluated, though increasing evidence supports their protective role against TB (82). Future studies should measure TBpV1-induced *Mtb*-specific antibodies and determine their correlation with protection. Finally, protection was tested in a single mouse genotype (BALB/c), though we have previously demonstrated the efficacy of rPICV-vectored TB vaccines in C57BL/6 mice (25). Future studies should extend these findings to different mouse models, including the highly susceptible C3HeB/FeJ mouse model and the guinea pig model (83–85).

In summary, the findings from this study demonstrate the potential of TBpV1 as both a prophylactic preventative and post-exposure treatment for adult pulmonary TB. These results support the continued development of rP18tri-vectored TB vaccines and highlight the potential of pooled, multi-antigen strategies to improve TB vaccine efficacy.

## Data Availability

The original contributions presented in the study are included in the article/**Supplementary Material**. Further inquiries can be directed to the corresponding author.

## Author’s note

The rP18tri vector described in this manuscript is the subject of an approved patent No. PCT/US2015/051337.

## Supplementary material

Supplementary Figure 1 Gating strategy for Ag85B/MHC-II tetramer-positive CD4 T cells (**A**) and EsxH/MHC-I tetramer-positive CD8 T cells (**B**).

Supplementary Figure 2 Gating strategy for the intracellular cytokine staining of double-positive Th1 CD4 T cells (**A**) and IL-17-positive Th17 Cells (**B**).
