# Supplementary material for: Prophylactic and post-exposure efficacy of a Pichinde virus vector-based tuberculosis vaccine
Source: Front Immunol. 2026 Jun 30;17:1861052. doi: 10.3389/fimmu.2026.1861052 (PMC13365046; doi:10.3389/fimmu.2026.1861052)
Supplement: Supplementary Figure 1 — Gating strategy for Ag85B/MHC-II tetramer-positive CD4 T cells (A) and EsxH/MHC-I tetramer-positive CD8 T cells (B). [file Presentation1.pptx]

## Slide 1
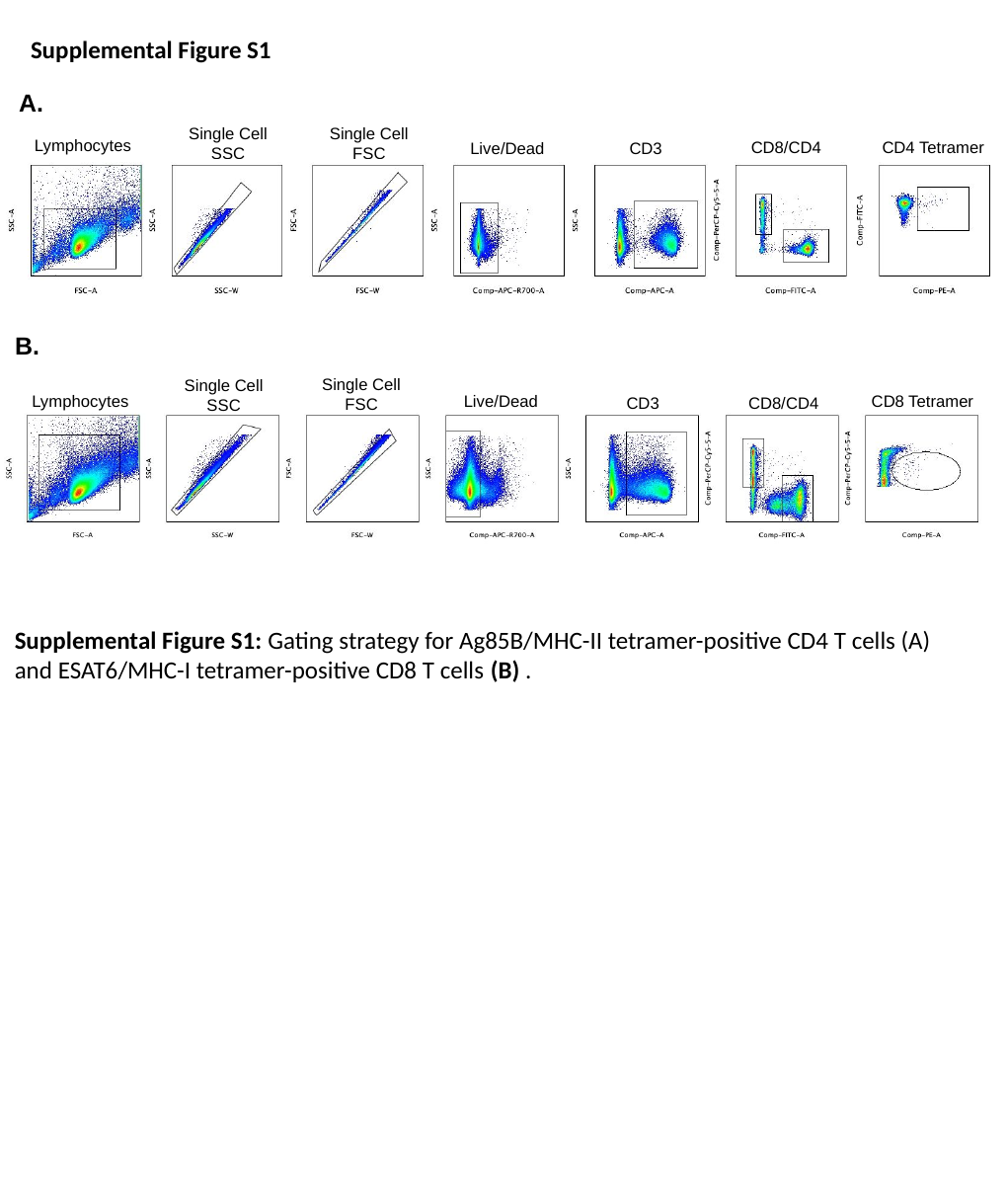

Supplemental Figure S1
A.
Single Cell SSC
Single Cell FSC
Lymphocytes
CD8/CD4
CD4 Tetramer
CD3
Live/Dead
B.
Single Cell FSC
Single Cell SSC
Live/Dead
CD8 Tetramer
Lymphocytes
CD3
CD8/CD4
Supplemental Figure S1: Gating strategy for Ag85B/MHC-II tetramer-positive CD4 T cells (A) and ESAT6/MHC-I tetramer-positive CD8 T cells (B) .

## Slide 2
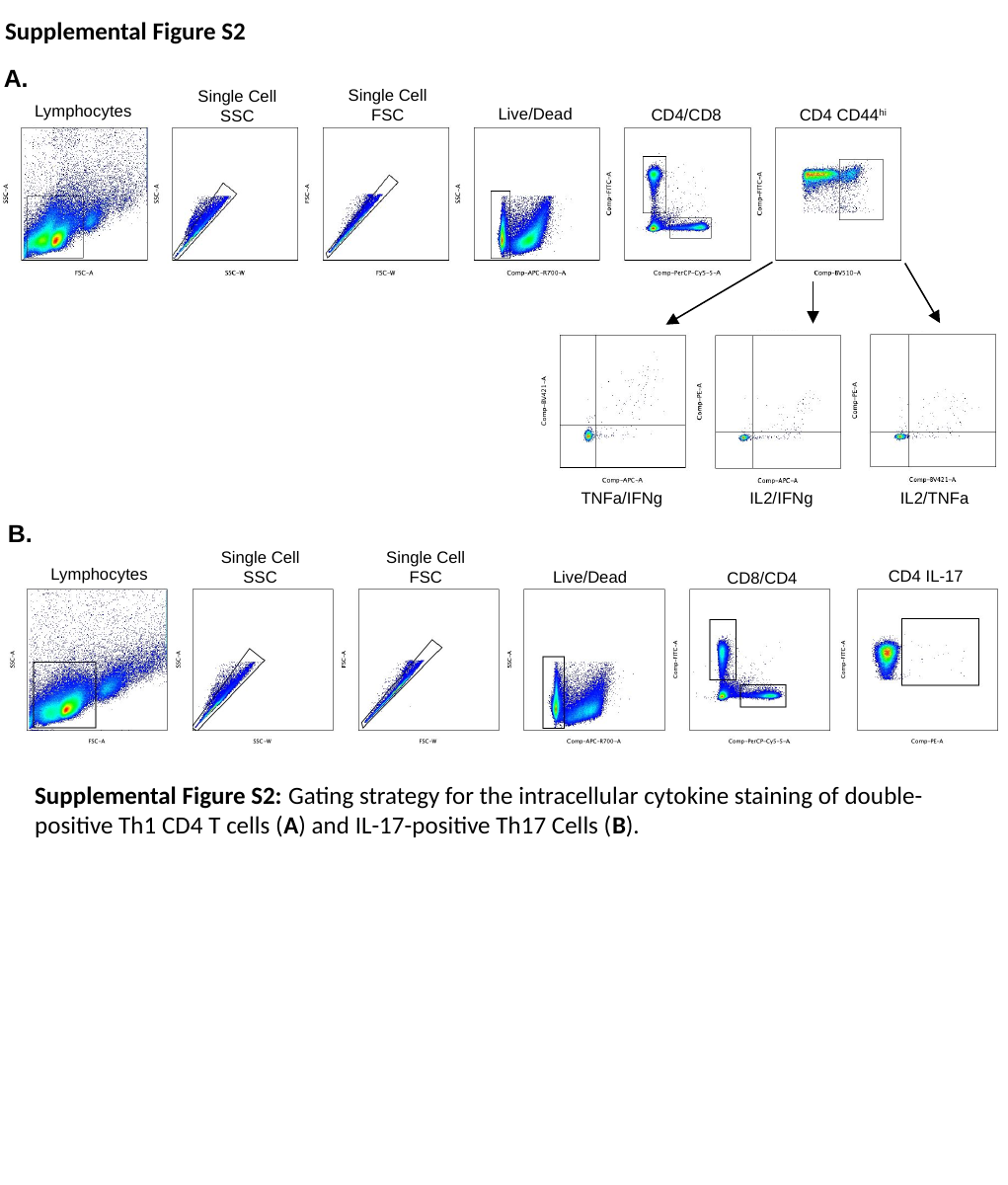

Supplemental Figure S2
A.
Single Cell FSC
Single Cell SSC
Lymphocytes
Live/Dead
CD4/CD8
CD4 CD44hi
TNFa/IFNg
IL2/IFNg
IL2/TNFa
B.
Single Cell SSC
Single Cell FSC
Lymphocytes
CD4 IL-17
Live/Dead
CD8/CD4
Supplemental Figure S2: Gating strategy for the intracellular cytokine staining of double-positive Th1 CD4 T cells (A) and IL-17-positive Th17 Cells (B).
